# Supplementary material for: 3,4-Enhanced Polymerization of Isoprene Catalyzed by Side-Arm Tridentate Iminopyridine Iron Complex with High Activity: Optimization via Response Surface Methodology
Source: Polymers (Basel). 2023 Feb 28;15(5):1231. doi: 10.3390/polym15051231 (PMC10007258; doi:10.3390/polym15051231)
Supplement: Supplementary file 1 [file polymers-15-01231-s001.zip › polymers-2214012-supplementary.pdf]

Supporting Information

3,4-Enhanced Polymerization of Isoprene Catalyzed by Side Arm Tridentate Iminopyridine Iron Complex with High Activity: Optimization via Response Surface Methodology

Total number of pages: 17

Total number of Figures: 32 (Figures S1-S32)

Table of Contents

1. NMR spectra of ligand and the representative polyisoprene .. 2

2. GPC of the Representative Polyisoprene 12

1. NMR spectra of ligand and the representative polyisoprene

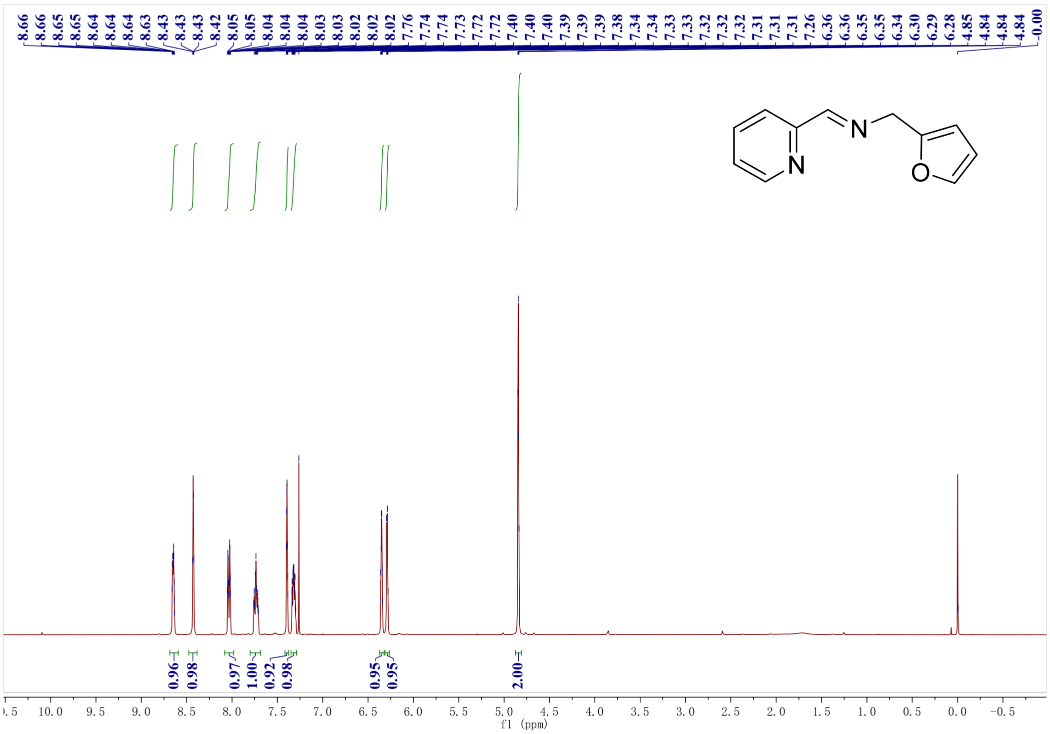

Figure S1. ^{1}H NMR spectrum (400 MHz, $CDCl_3$, 298 K) of **L1**

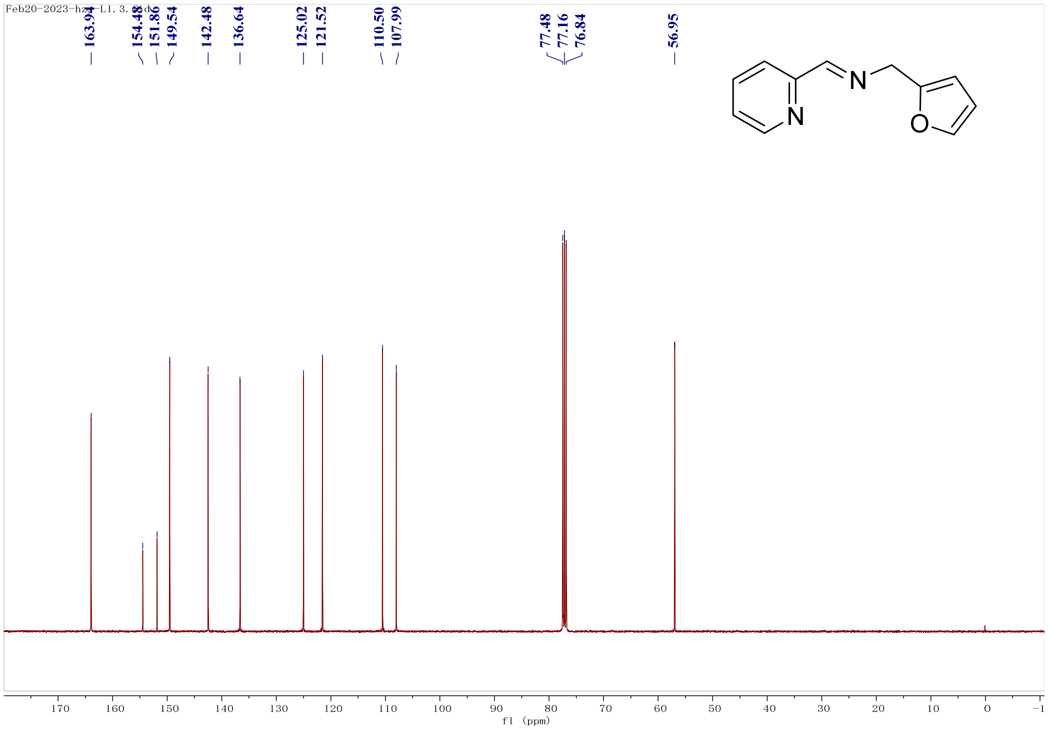

Figure S2. ^{13}C NMR spectrum (100 MHz, $CDCl_3$, 298 K) of **L 1**

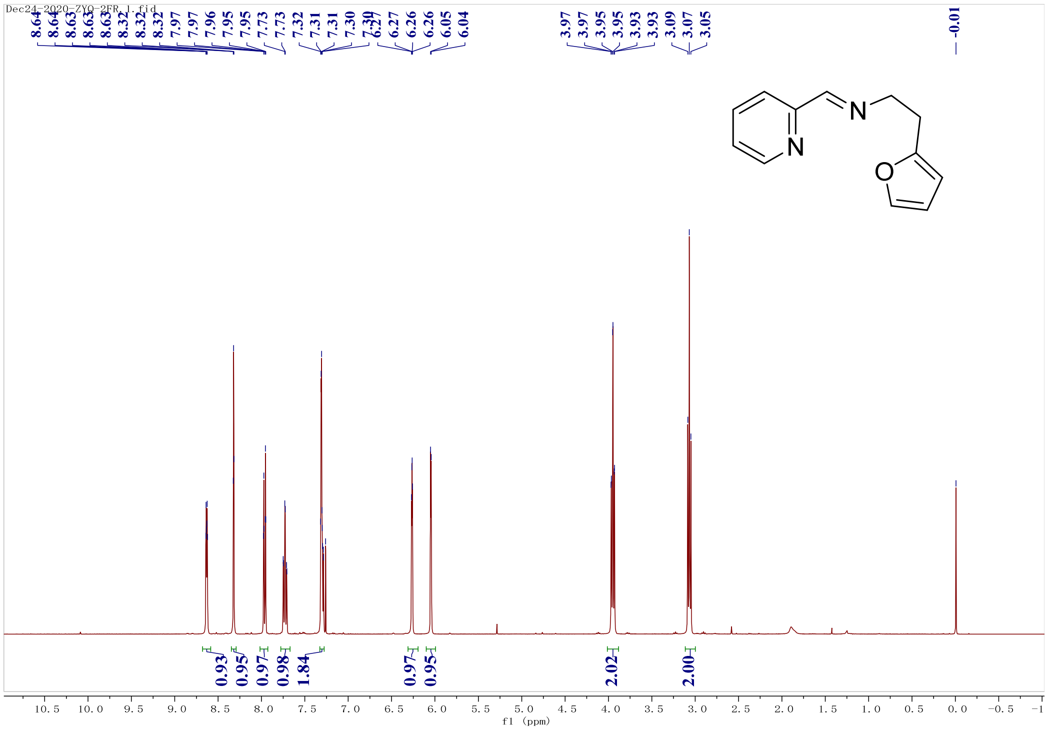

Figure S3. ^{1}H NMR spectrum (400 MHz, $CDCl_3$, 298 K) of **L 2**

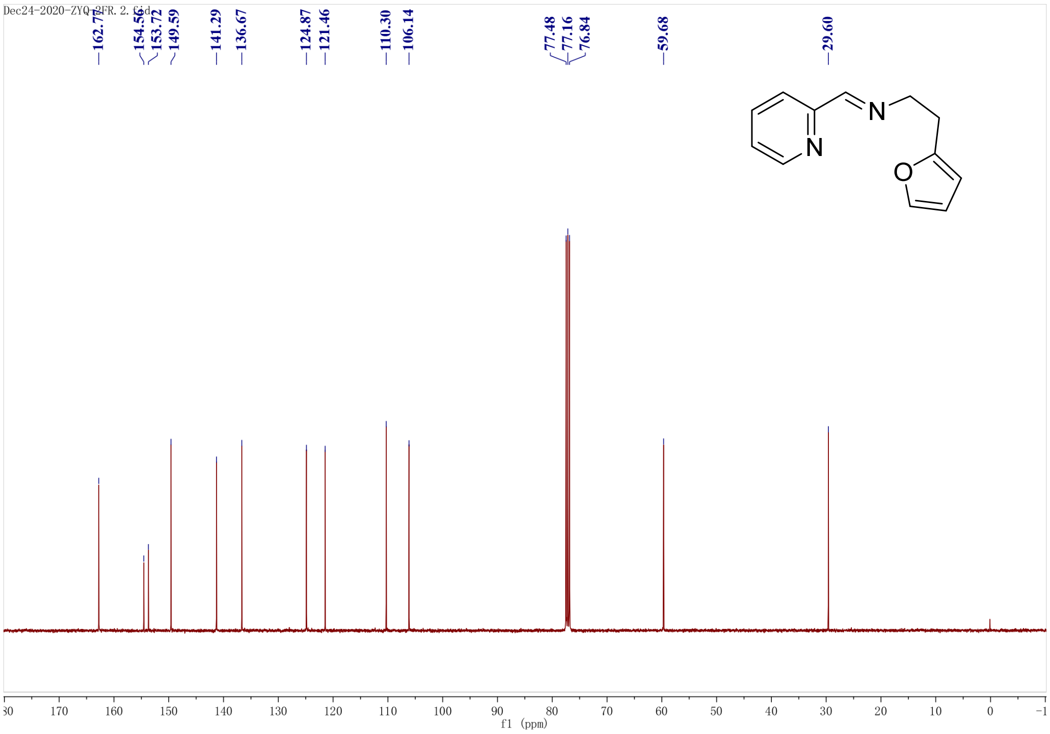

Figure S4. ^{13}C NMR spectrum (100 MHz, $CDCl_3$, 298 K) of **L 2**

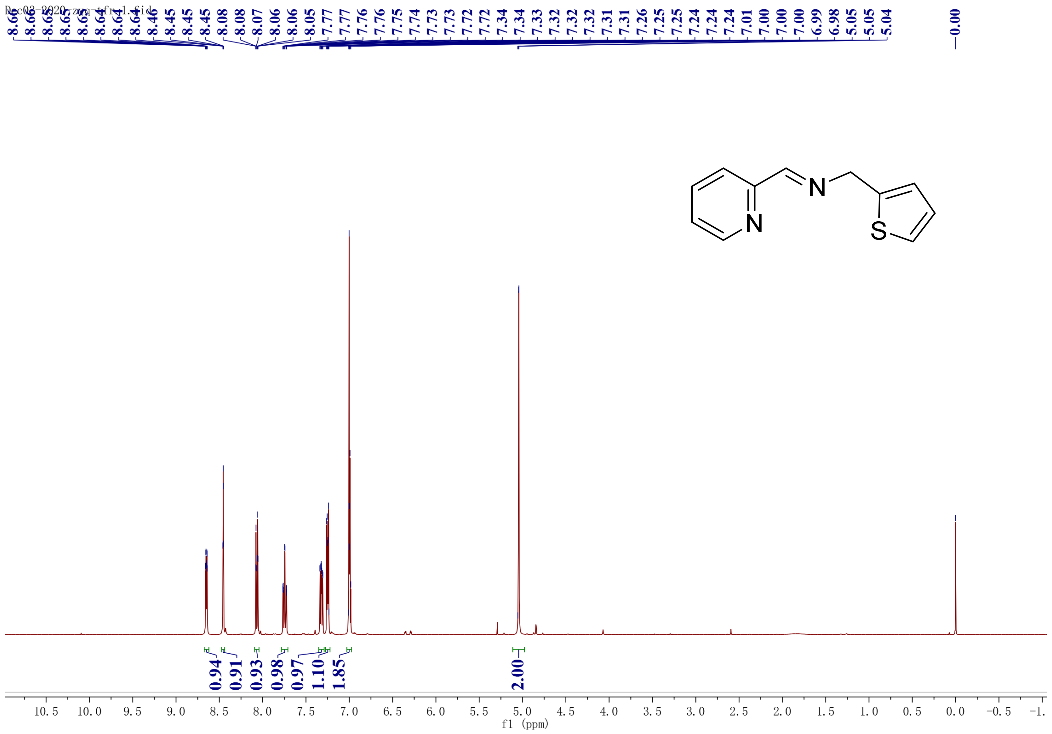

Figure S5. ^{1}H NMR spectrum (400 MHz, $CDCl_3$, 298 K) of **L 3**

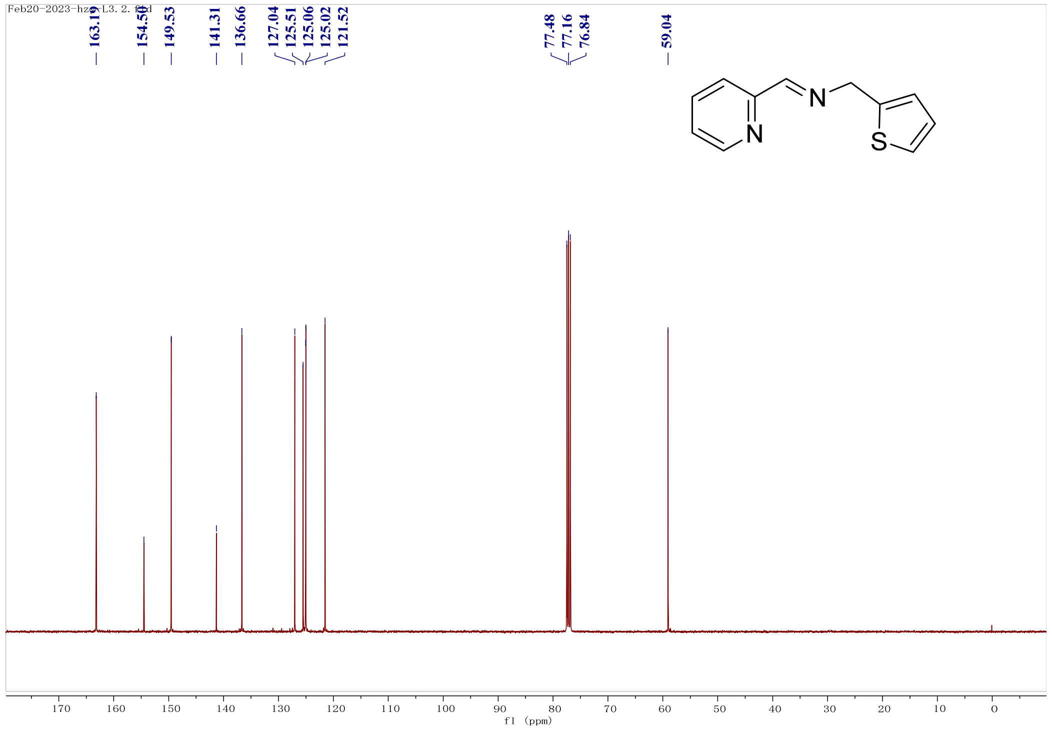

Figure S6. ^{13}C NMR spectrum (100 MHz, $CDCl_3$, 298 K) of **L 3**

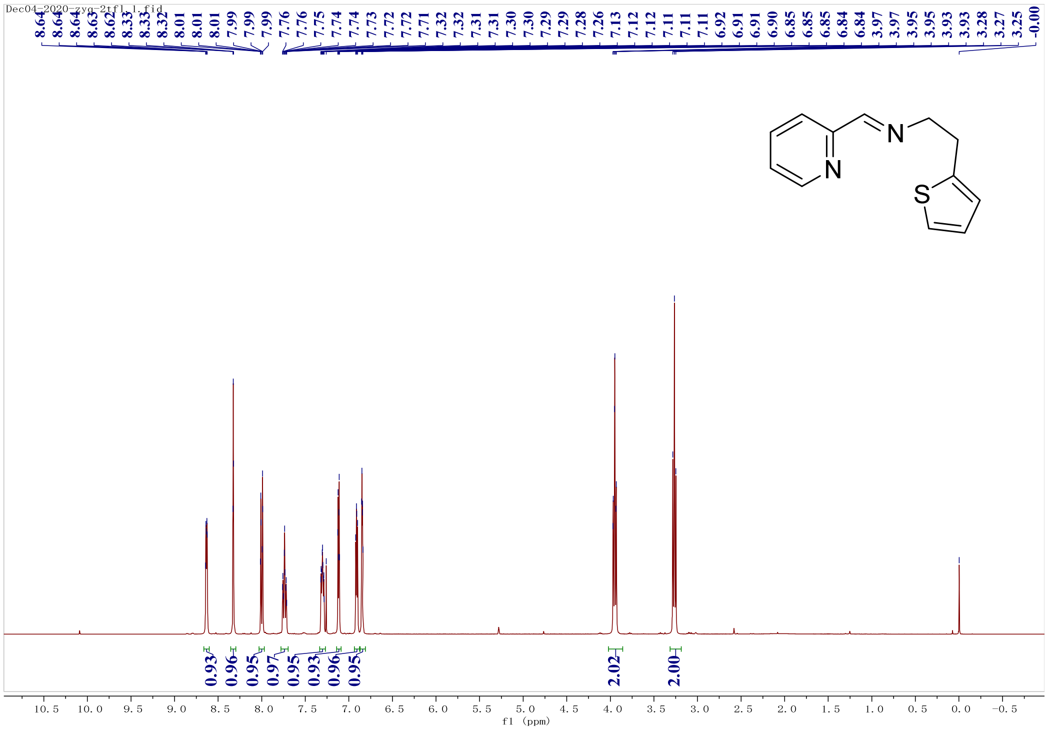

Figure S7. ^{1}H NMR spectrum (400 MHz, $CDCl_3$, 298 K) of **L 4**

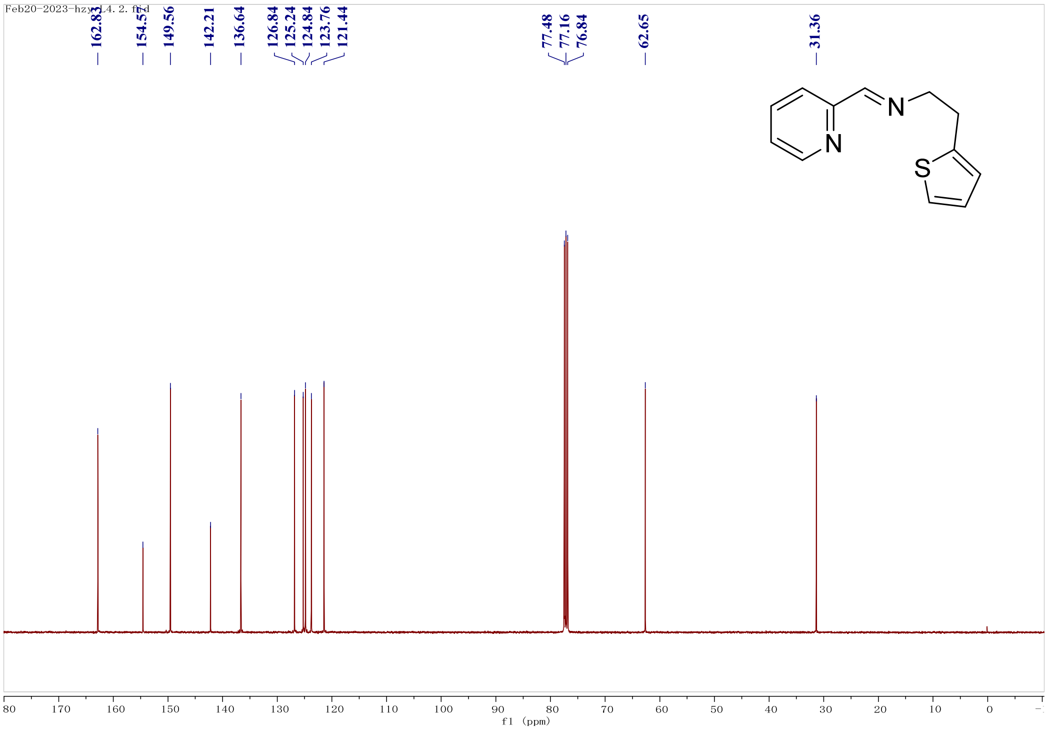

Figure S8. ^{13}C NMR spectrum (100 MHz, $CDCl_3$, 298 K) of **L 4**

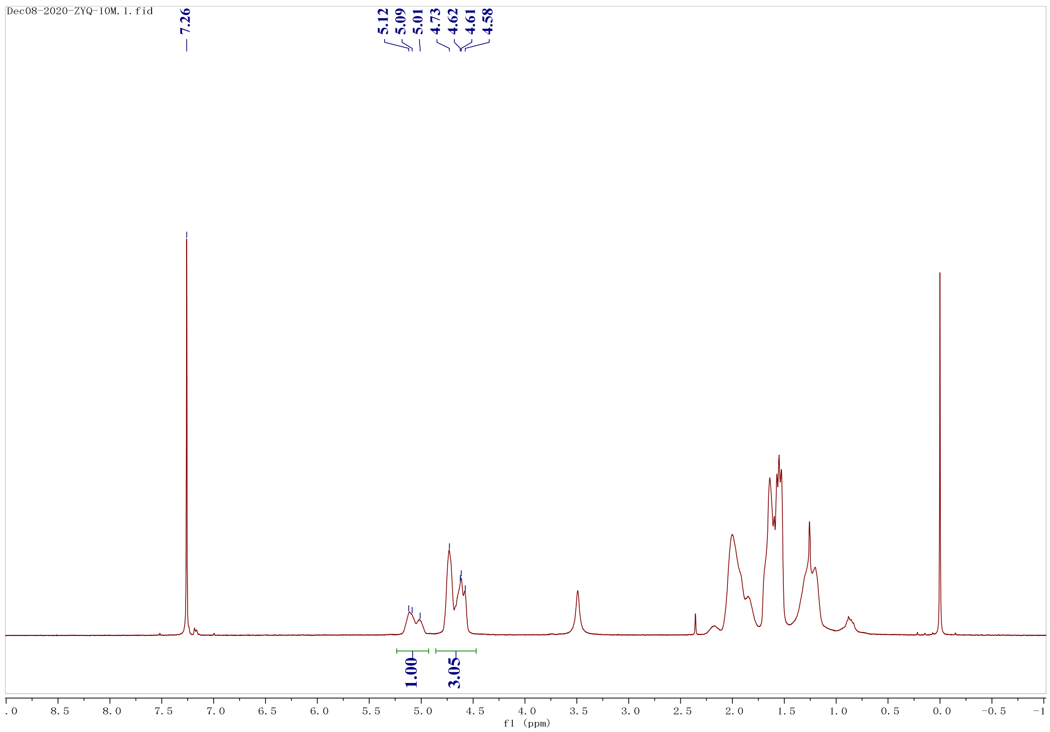

Figure S9 ^{1}H NMR spectrum (400 MHz, $CDCl_3$, 298 K) of polymer (Table 1 entry 1)

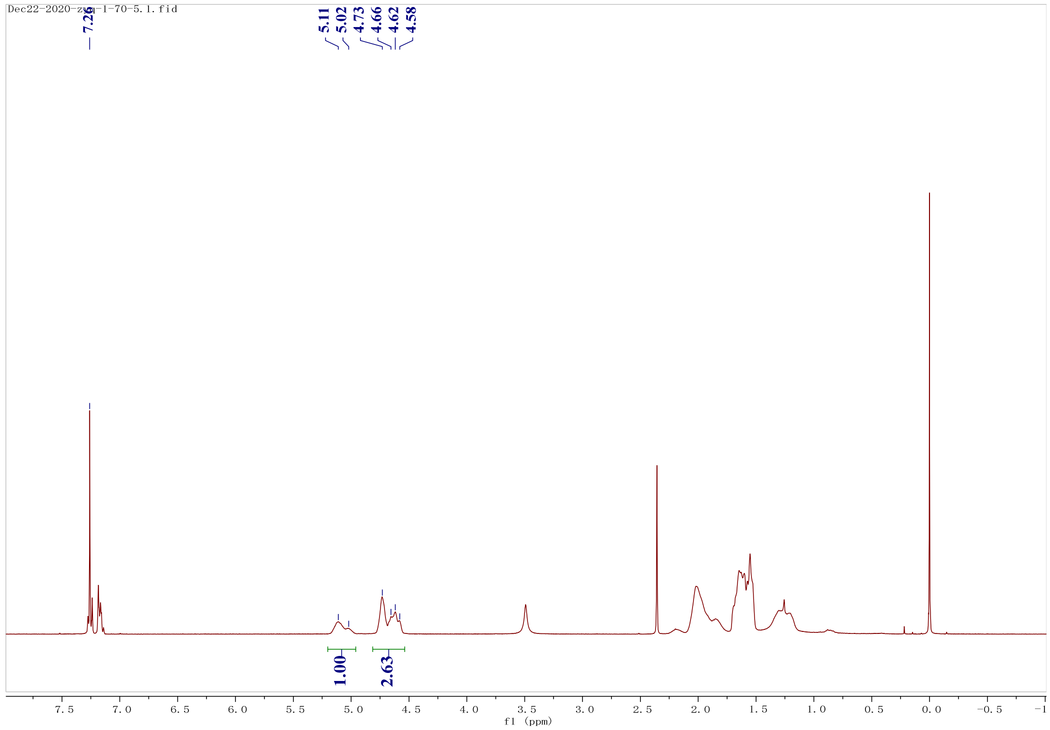

Figure S10 ^{1}H NMR spectrum (400 MHz, $CDCl_3$, 298 K) of polymer (Table 1 entry 2)

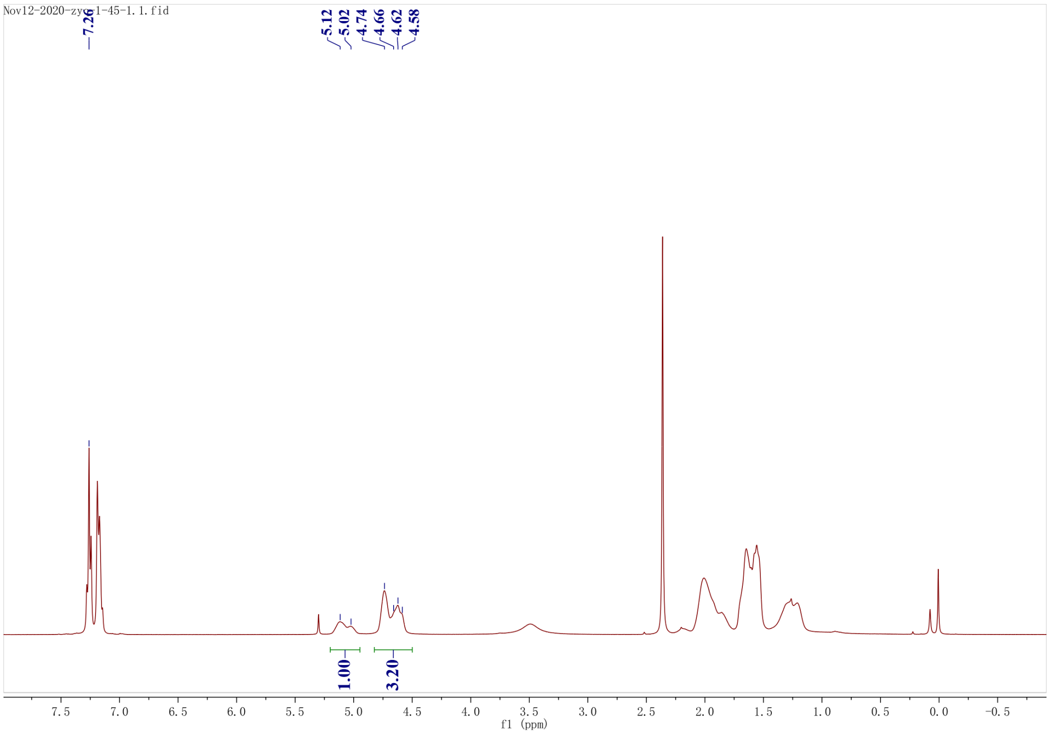

Figure S11 ^{1}H NMR spectrum (400 MHz, $CDCl_3$, 298 K) of polymer (Table 1 entry 3)

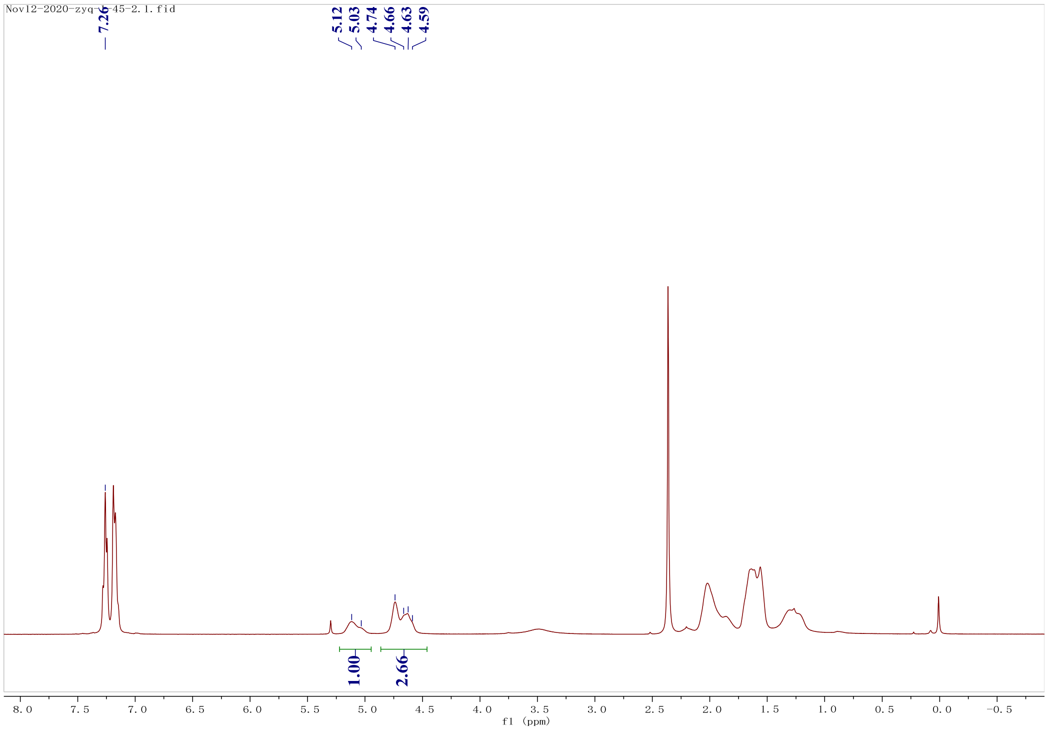

Figure S12 ^{1}H NMR spectrum (400 MHz, $CDCl_3$, 298 K) of polymer (Table 1 entry 4)

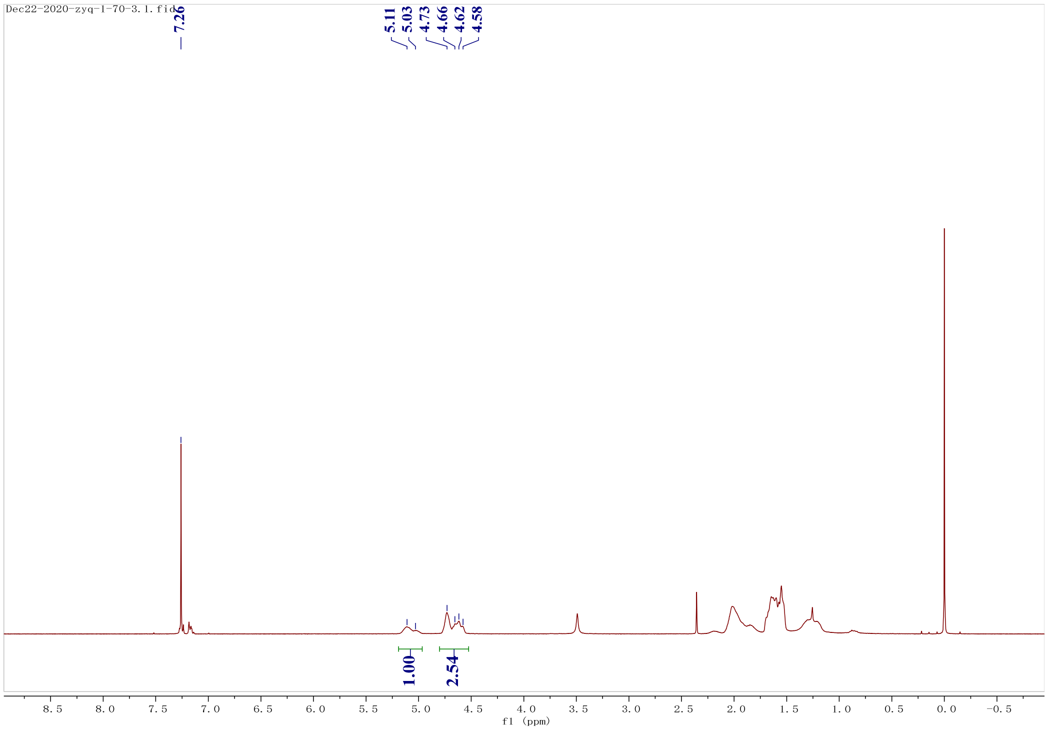

Figure S13 ^{1}H NMR spectrum (400 MHz, $CDCl_3$, 298 K) of polymer (Table 2 Entry 2)

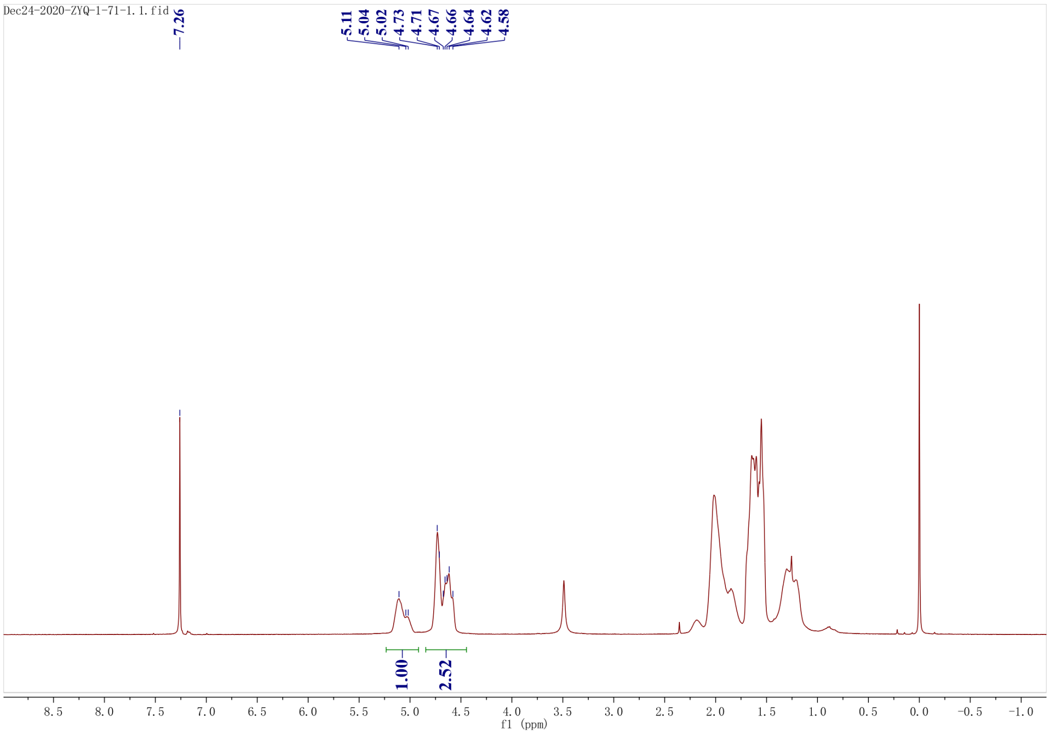

Figure S14 ^{1}H NMR spectrum (400 MHz, $CDCl_3$, 298 K) of polymer (Table 2 Entry 3)

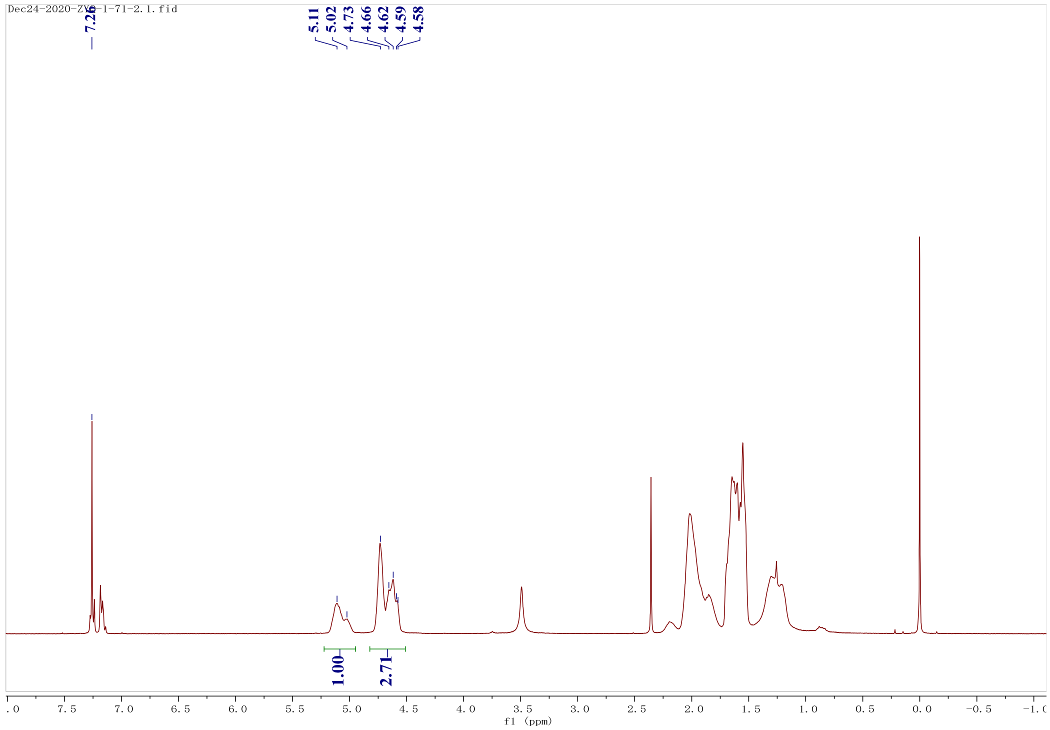

Figure S15 ^{1}H NMR spectrum (400 MHz, $CDCl_3$, 298 K) of polymer (Table 2 Entry 4)

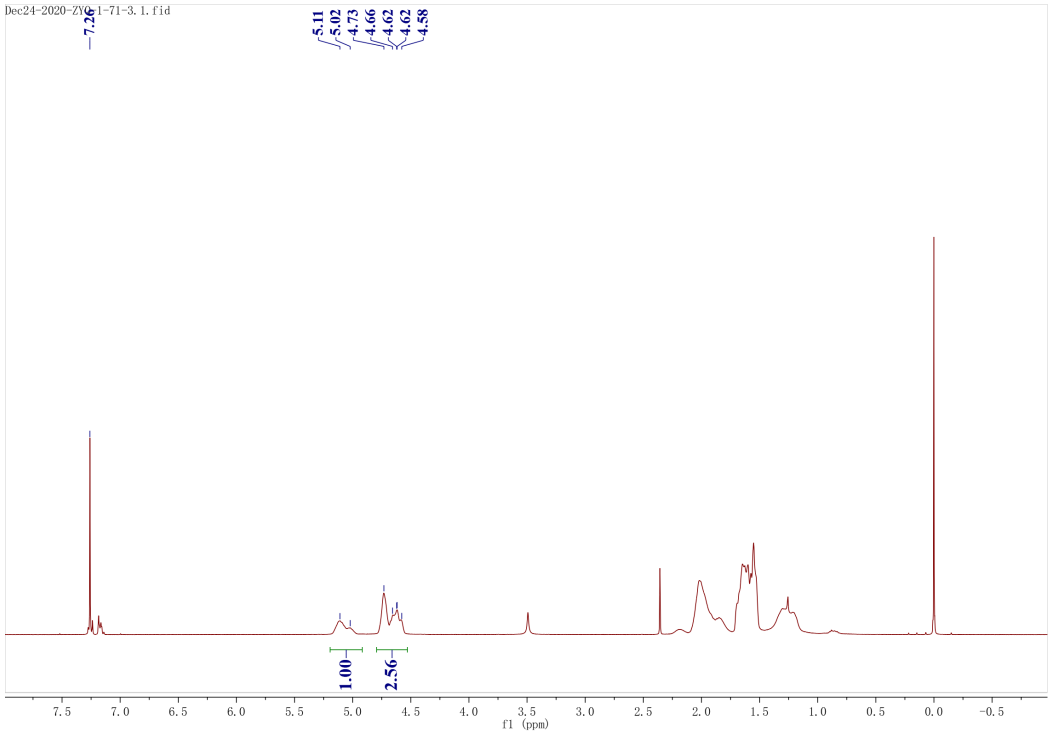

Figure S16 ^{1}H NMR spectrum (400 MHz, $CDCl_3$, 298 K) of polymer (Table 2 Entry 5)

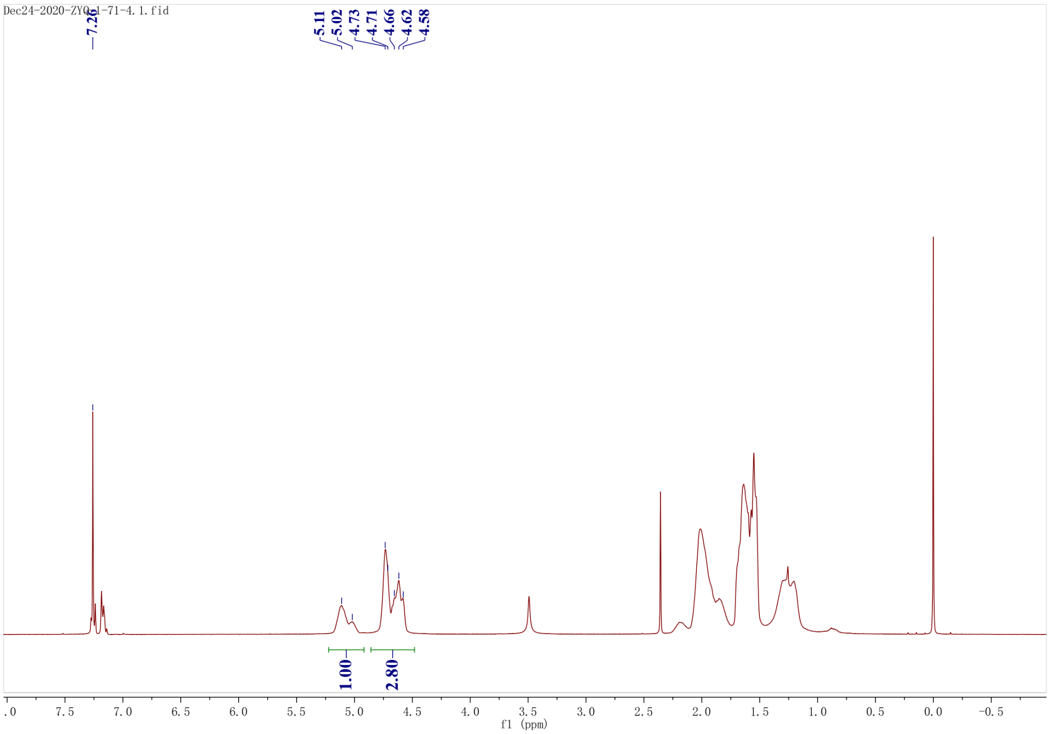

Figure S17 ^{1}H NMR spectrum (400 MHz, $CDCl_3$, 298 K) of polymer (Table 2 Entry 6)

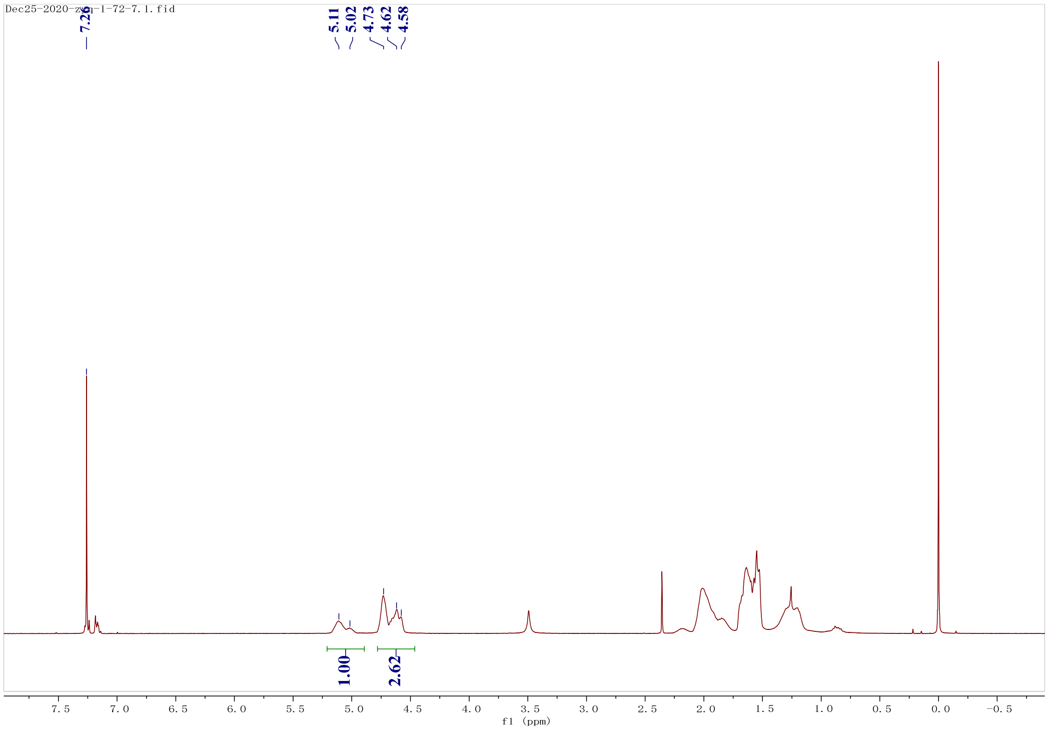

Figure S18 ^{1}H NMR spectrum (400 MHz, $CDCl_3$, 298 K) of polymer (Table 2 Entry 7)

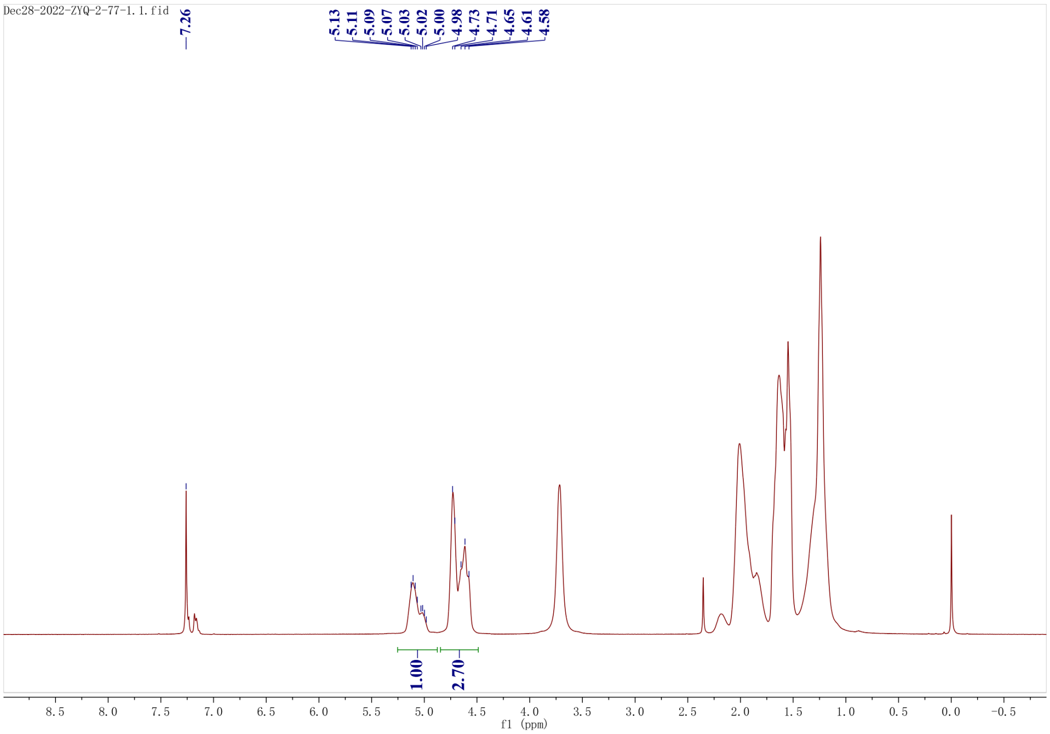

Figure S19 ^{1}H NMR spectrum (400 MHz, $CDCl_3$, 298 K) of polymer (Table 2 Entry 8)

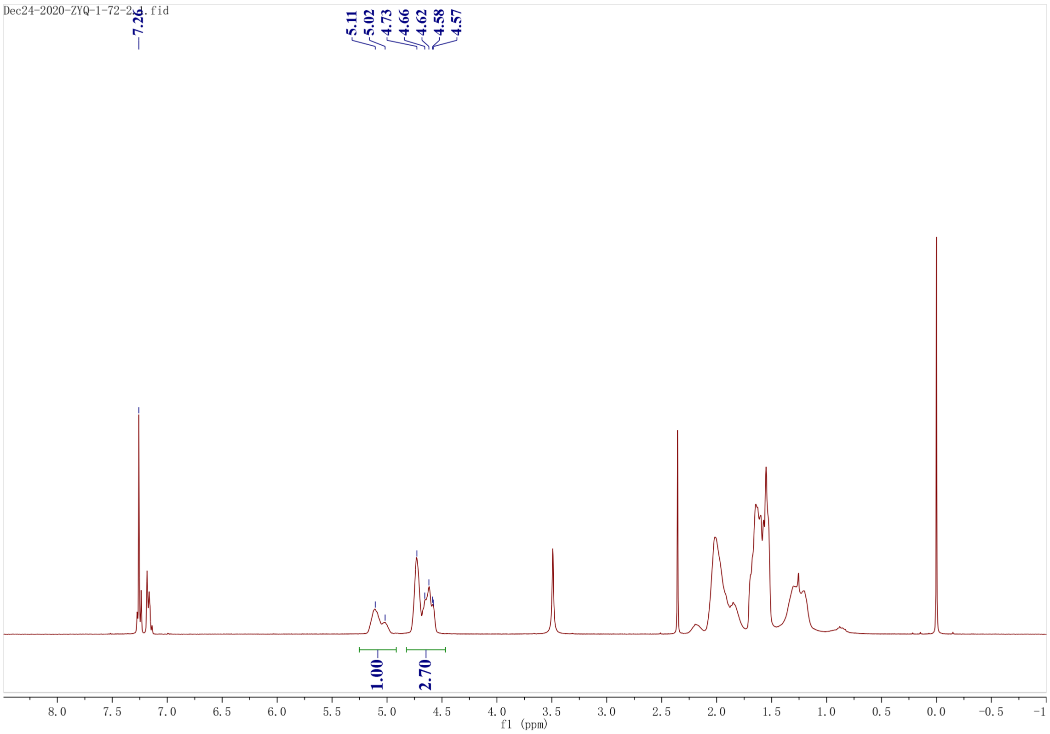

Figure S20 ^{1}H NMR spectrum (400 MHz, $CDCl_3$, 298 K) of polymer (Table 2 Entry 9)

2. GPC of the Representative Polyisoprene

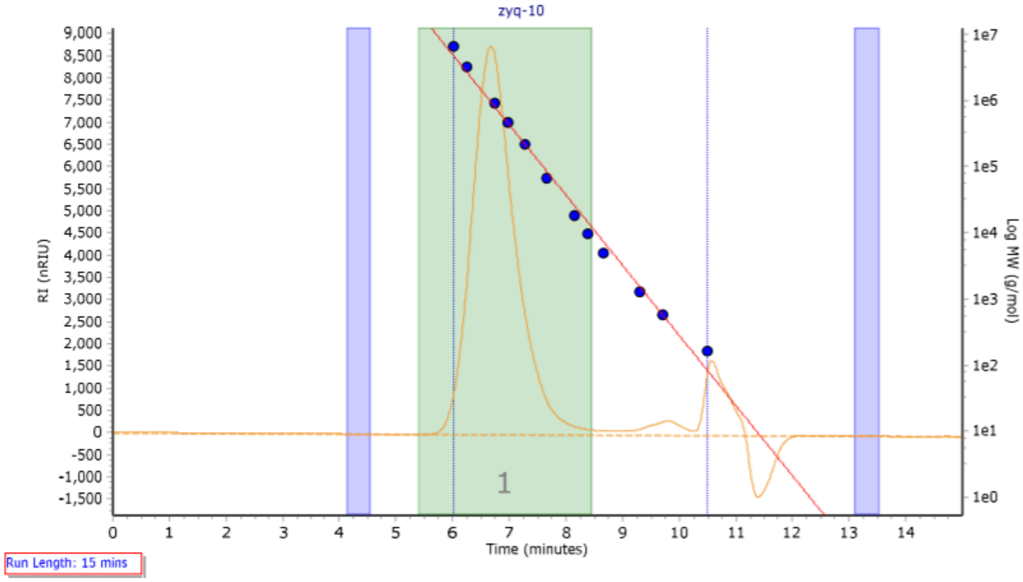

Molecular Weight Averages

Peak	Mp (g/mol)	Mn (g/mol)	Mw (g/mol)	Mz (g/mol)	Mz+1 (g/mol)	Mv (g/mol)	PD
Peak 1	922227	380223	1084155	2017165	3251716	1865578	2.851

Figure S21. GPC of Polyisoprene (Table 1 entry 1)

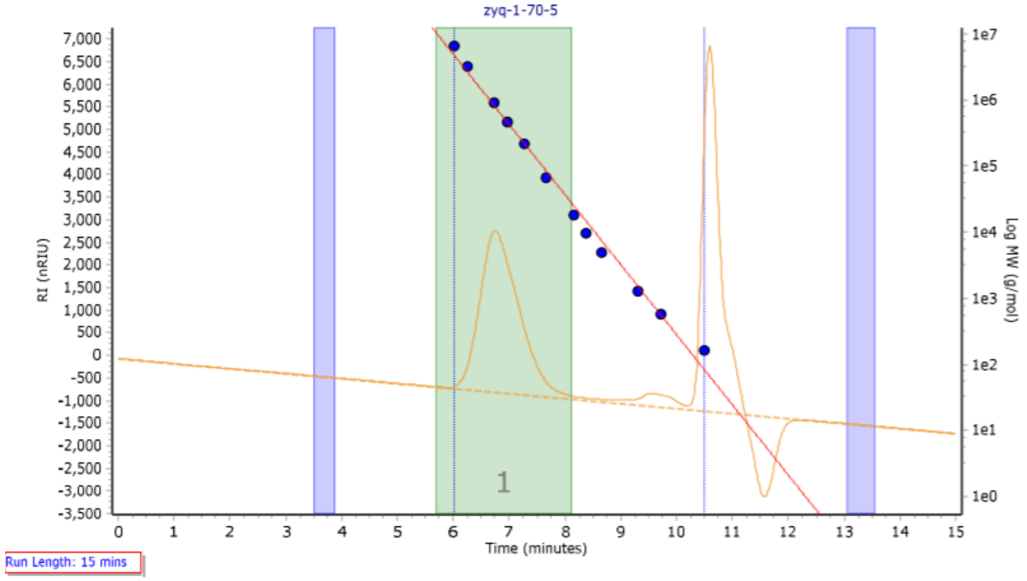

Molecular Weight Averages

Peak	Mp (g/mol)	Mn (g/mol)	Mw (g/mol)	Mz (g/mol)	Mz+1 (g/mol)	Mv (g/mol)	PD
Peak 1	783606	373705	780304	1306015	2016286	1220938	2.088

Figure S22. GPC of Polyisoprene (Table 1 entry 2)

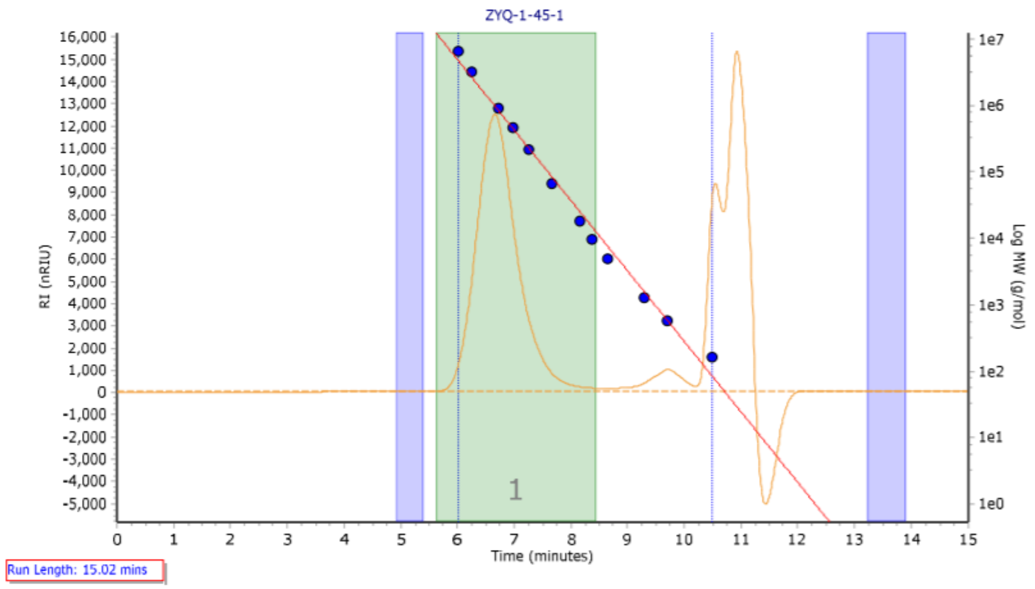

Molecular Weight Averages

Peak	Mp (g/mol)	Mn (g/mol)	Mw (g/mol)	Mz (g/mol)	Mz+1 (g/mol)	Mv (g/mol)	PD
Peak 1	960556	426845	1128803	1980576	3014916	1846123	2.645

Figure S23. GPC of Polyisoprene (Table 1 entry 3)

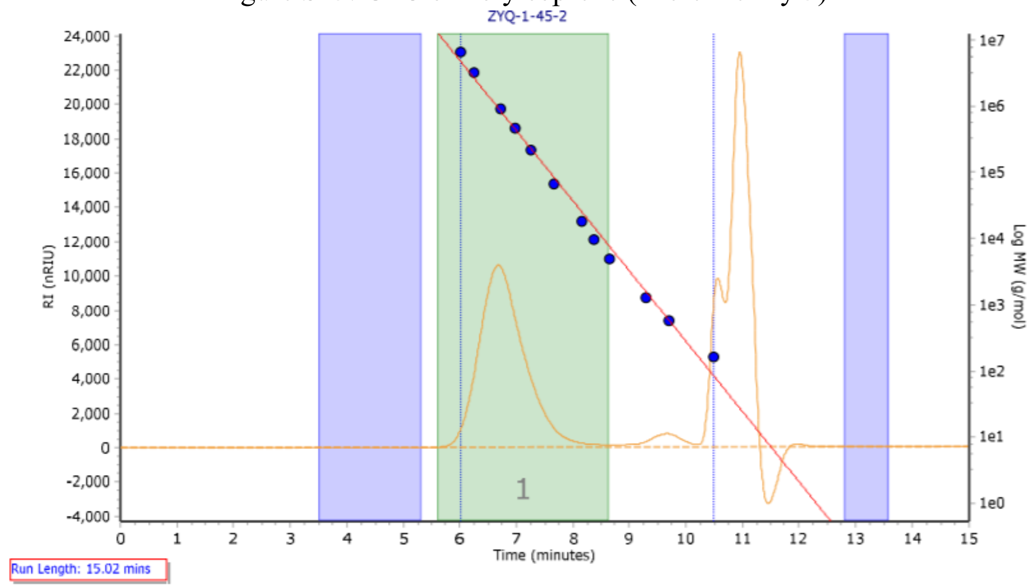

Molecular Weight Averages

Peak	Mp (g/mol)	Mn (g/mol)	Mw (g/mol)	Mz (g/mol)	Mz+1 (g/mol)	Mv (g/mol)	PD
Peak 1	903640	331697	1044012	1952181	3031516	1810857	3.147

Figure S24. GPC of Polyisoprene (Table 1 entry 4)

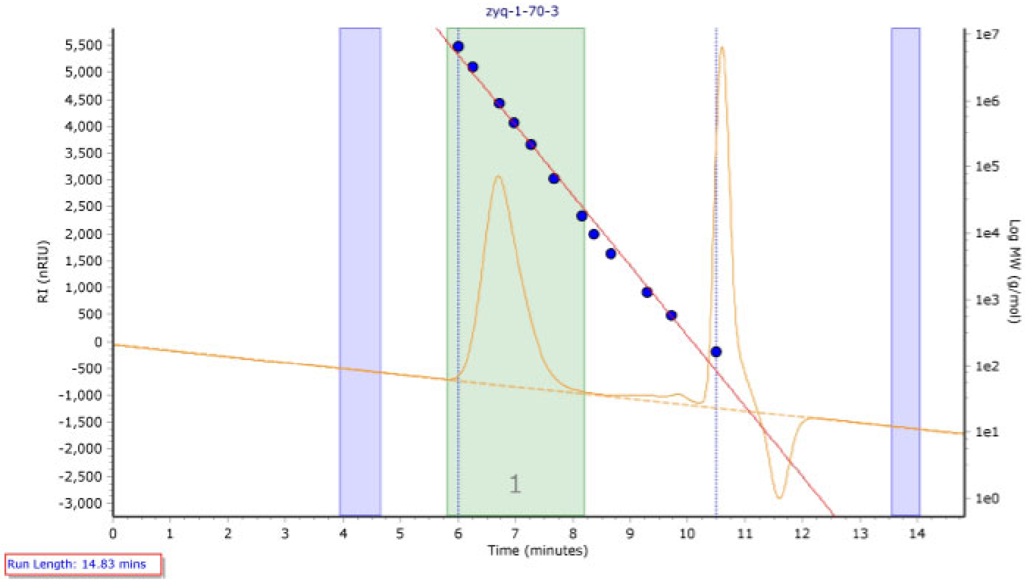

Molecular Weight Averages

Peak	Mp (g/mol)	Mn (g/mol)	Mw (g/mol)	Mz (g/mol)	Mz+1 (g/mol)	Mv (g/mol)	PD
Peak 1	850096	428655	897383	1469001	2138717	1380594	2.093

Figure S25. GPC of Polyisoprene (Table 2 entry 2)

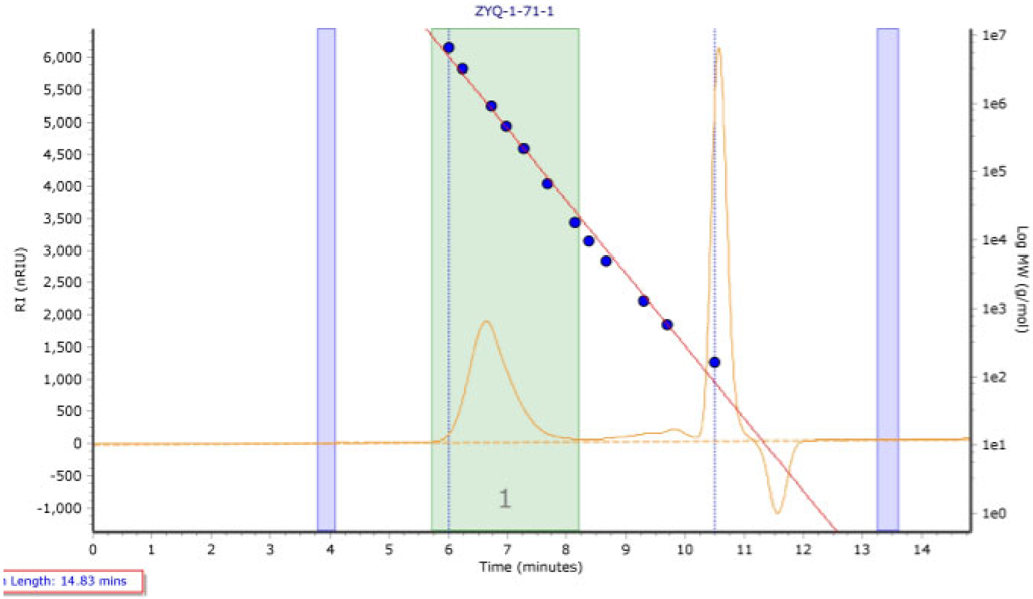

Molecular Weight Averages

Peak	Mp (g/mol)	Mn (g/mol)	Mw (g/mol)	Mz (g/mol)	Mz+1 (g/mol)	Mv (g/mol)	PD
Peak 1	1021058	448521	1079941	1865584	2844901	1741181	2.408

Figure S26. GPC of Polyisoprene (Table 2 entry 3)

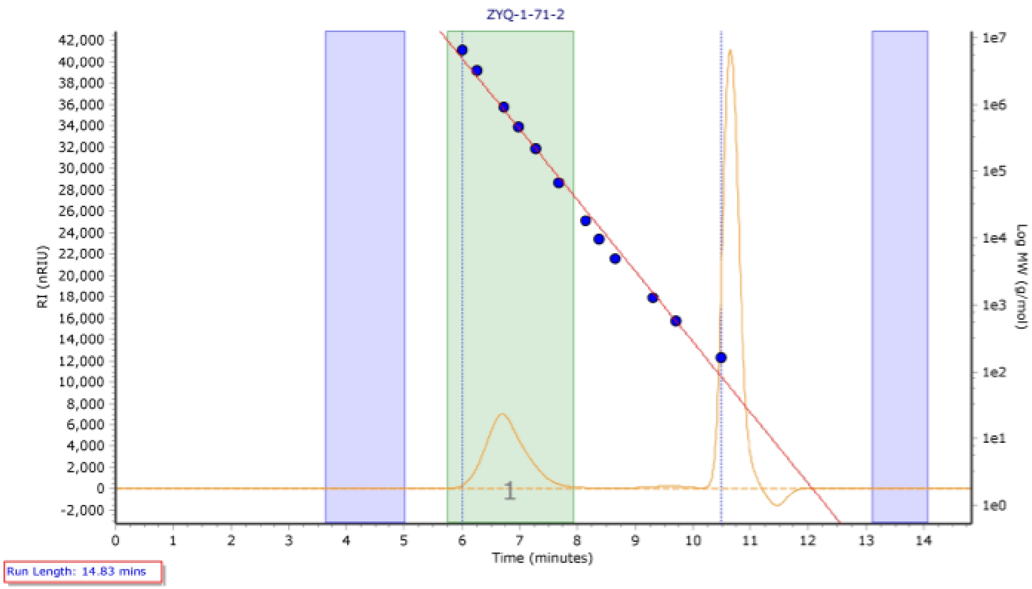

Molecular Weight Averages

Peak	Mp (g/mol)	Mn (g/mol)	Mw (g/mol)	Mz (g/mol)	Mz+1 (g/mol)	Mv (g/mol)	PD
Peak 1	885427	482750	978875	1657316	2518014	1547372	2.028

Figure S27. GPC of Polyisoprene (Table 2 entry 4)

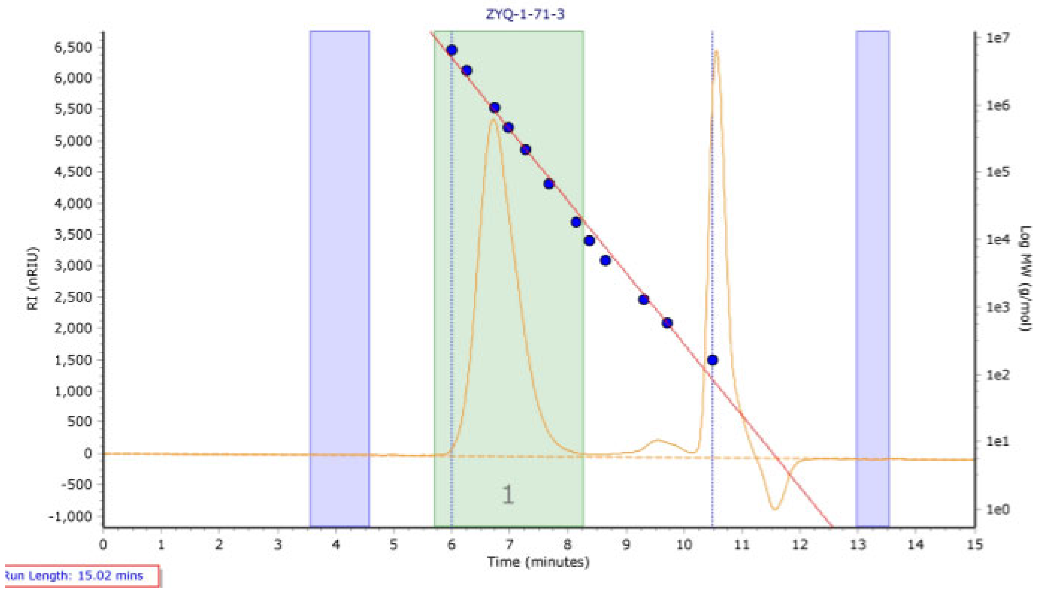

Molecular Weight Averages

Peak	Mp (g/mol)	Mn (g/mol)	Mw (g/mol)	Mz (g/mol)	Mz+1 (g/mol)	Mv (g/mol)	PD
Peak 1	832962	405036	870442	1456354	2203553	1362513	2.149

Figure S28. GPC of Polyisoprene (Table 2 entry 5)

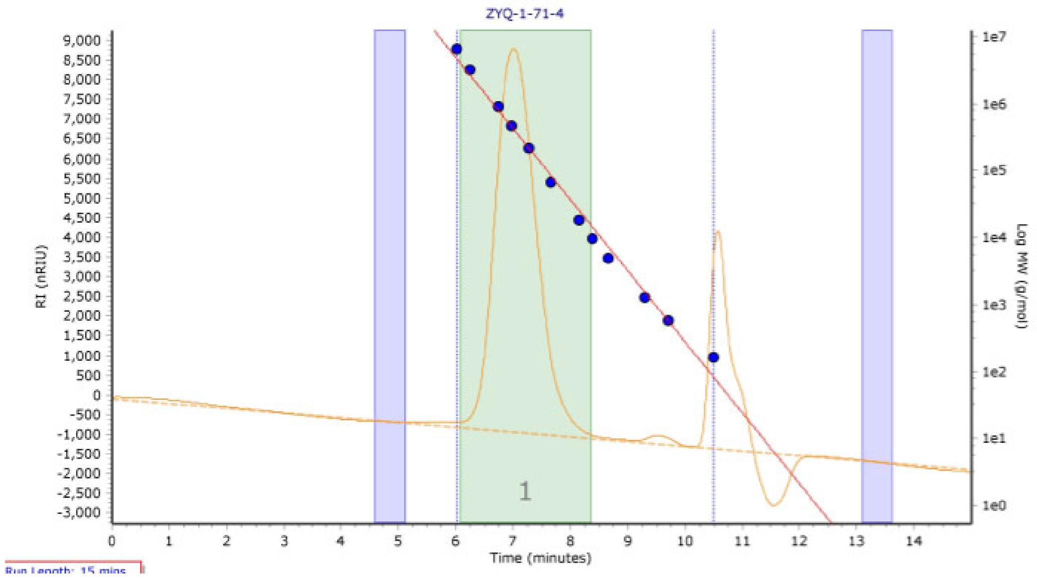

Molecular Weight Averages

Peak	Mp (g/mol)	Mn (g/mol)	Mw (g/mol)	Mz (g/mol)	Mz+1 (g/mol)	Mv (g/mol)	PD
Peak 1	408449	233656	471091	802294	1255377	746754	2.016

Figure S29. GPC of Polyisoprene (Table 2 entry 6)

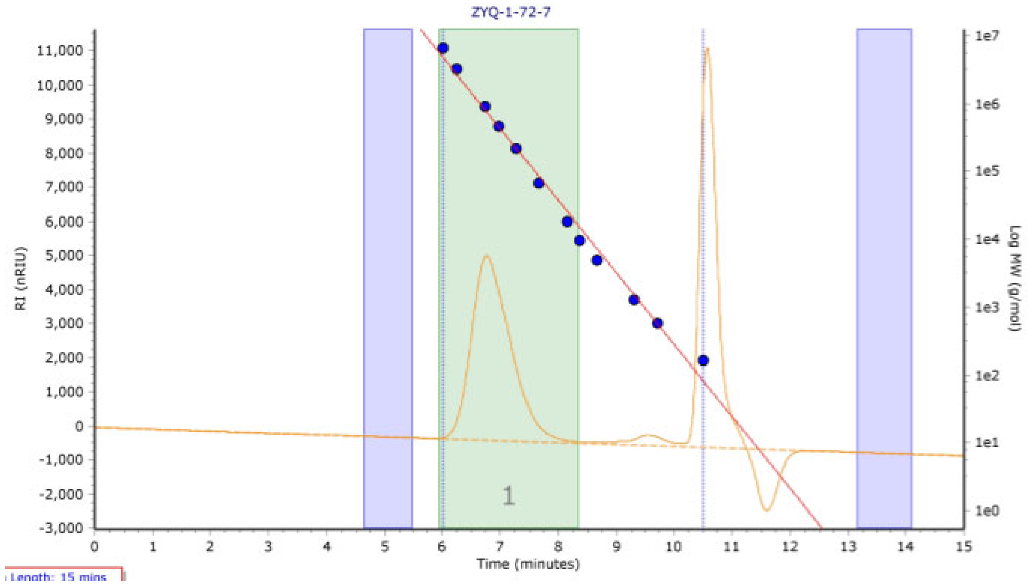

Molecular Weight Averages

Peak	Mp (g/mol)	Mn (g/mol)	Mw (g/mol)	Mz (g/mol)	Mz+1 (g/mol)	Mv (g/mol)	PD
Peak 1	752338	355169	733539	1164617	1647078	1099570	2.065

Figure S30. GPC of Polyisoprene (Table 2 entry 7)

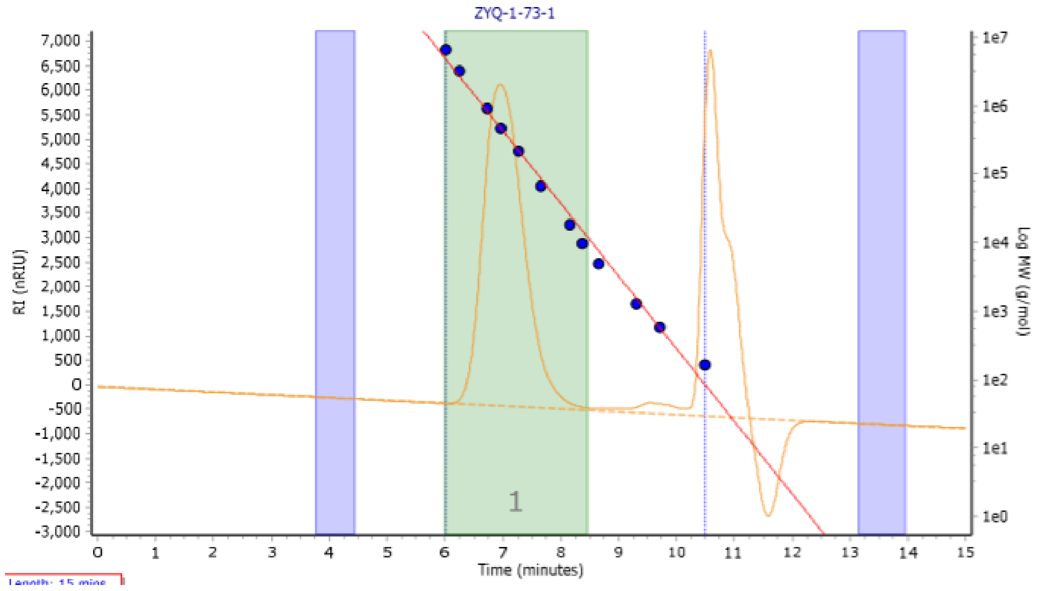

Molecular Weight Averages

Peak	Mp (g/mol)	Mn (g/mol)	Mw (g/mol)	Mz (g/mol)	Mz+1 (g/mol)	Mv (g/mol)	PD
Peak 1	471016	251519	494796	775009	1080903	733082	1.967

Figure S31. GPC of Polyisoprene (Table 2 entry 8)

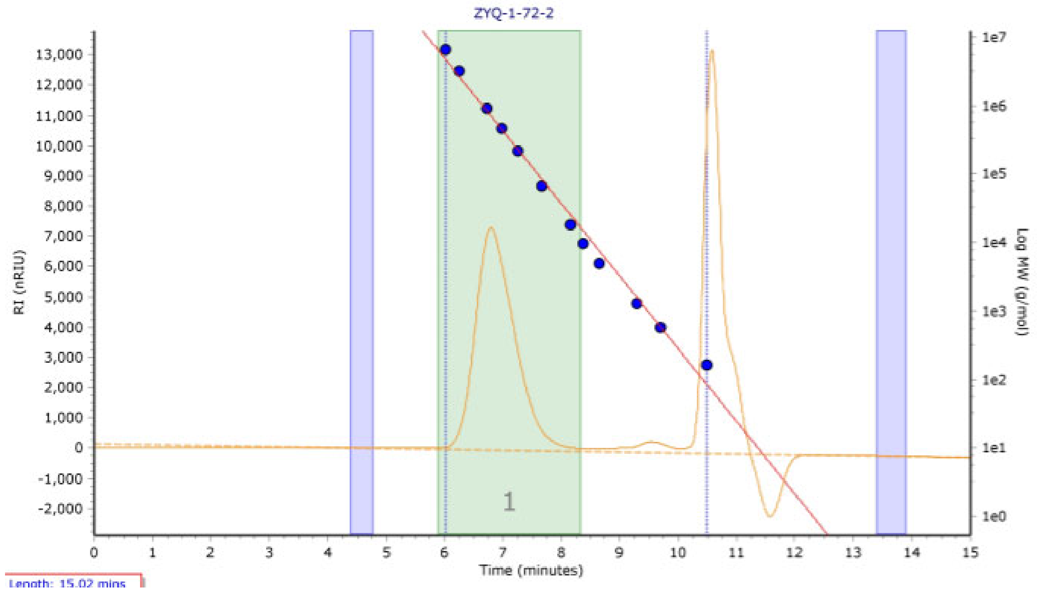

Molecular Weight Averages

Peak	Mp (g/mol)	Mn (g/mol)	Mw (g/mol)	Mz (g/mol)	Mz+1 (g/mol)	Mv (g/mol)	PD
Peak 1	693494	334477	694523	1123405	1674722	1054930	2.076

Figure S32. GPC of Polyisoprene (Table 2 entry 9)
